# Vascularization of the trachea in the bottlenose dolphin: comparison with bovine and evidence for evolutionary adaptations to diving

**DOI:** 10.1098/rsos.171645

**Published:** 2018-04-18

**Authors:** Cristina Ballarin, Paola Bagnoli, Antonella Peruffo, Bruno Cozzi

**Affiliations:** 1 Department of Comparative Biomedicine and Food Science, University of Padova, viale dell'Università 16, 35020, Legnaro, PD, Italy; 2 Technology Transfer Office, Politecnico di Milano, Milan, Italy

**Keywords:** trachea, bottlenose dolphin, vascular lacunae, valve-like structure

## Abstract

The rigid structure of the mammalian trachea is functional to maintain constant patency and airflow during breathing, but no gas exchange takes place through its walls. The structure of the organ in dolphins shows increased rigidity of the tracheal cartilaginous rings and the presence of vascular lacunae in the submucosa. However, no actual comparison was ever made between the size and capacity of the vascular lacunae of the dolphin trachea and the potentially homologous structures of terrestrial mammals. In the present study, the extension of the lacunae has been compared between the bottlenose dolphin and the bovine, a closely related terrestrial Cetartiodactyla. Our results indicate that the extension of the blood spaces in the submucosa of dolphins is over 12 times larger than in the corresponding structure of the bovines. Furthermore, a microscopic analysis revealed the presence of valve-like structures in the walls of the cetacean lacunae. The huge difference in size suggests that the lacunae are not merely a product of individual physiological plasticity, but may constitute a true adaptive evolutionary character, functional to life in the aquatic environment. The presence of valve-like structures may be related to the regulation of blood flow, and curtail excessive compression under baric stress at depth.

## 1. Introduction

The trachea is a major dead space in the respiratory apparatus, as no gas exchange occurs through its walls [1]. The structure of the trachea in the bottlenose dolphin, as in the majority of marine Cetartiodactyla, shows clear signs of adaptation to aquatic life: reduced length following shortening of the neck, wide diameter, thick walls with continuous cartilaginous rings are functional to rapid gas exchanges at the surface in mammals that have extensive fusion of the cervical vertebrae and virtually no visible neck [2]. The spiralling, and sometimes continuous cartilaginous rings, are devoid of the dorsal open ends and, consequently, show no *musculus trachealis* proper. Such structural organization improves rigidity of the organ in dolphins, in contrast with terrestrial mammals, including Cetartiodactyla as the bovine and Perissodactyla as the horse, in which the *m. trachealis* is short and attached close to, and inside, the opening of each ring. As a further comparison, the large *m. trachealis* constitutes the dorsal wall of the trachea in humans and goats.

Furthermore, the submucosa of the trachea of the striped dolphin [3], bottlenose dolphin [4] and pygmy sperm whale [5], contains large and conspicuous blood-filled venous spaces or vascular lacunae. The presence of extensive visceral innervation, at least in the striped dolphin [3], may be related to the filling of the lacunae. If that is the case, then the lacunae may thus be considered part of an erectile tissue (for details and review on the innervation of the mammalian trachea see [6]).

The presence of an extensive net of vascular lacunae within the hard walls of a respiratory dead space constitutes an apparent paradox in a diving mammal, especially during the (potentially rapid) ascent to the surface. During ascent, the expanding gas (air) might exit the vascular lacunae and thus create the conditions for potential breath-holding decompression sickness, which appears counter-intuitive if we consider the specific morphology of the cetacean respiratory apparatus as the result of evolutionary pressure. In fact, there is at present no solid ground to regard the vascular lacunae in the dolphin trachea as the product of an independent evolutionary trait, because no direct comparison has ever been made between the vascular lacunae of the dolphin trachea and the corresponding structures of terrestrial Cetartiodactyla. The presence and extension of the blood spaces in the lamina propria and submucosa of the cetacean trachea could be the product of evolutionary selection and adaptation. In the present study, we contribute by comparing the morphometry of these structures in the organs of bovines and bottlenose dolphins.

## 2. Material and methods

### 2.1. Tracheas and histological procedures

For the present study, we compared the tracheas of four bottlenose dolphins *Tursiops truncatus* and two bovines *Bos taurus*. The tracheas of the dolphins were obtained from the Mediterranean marine mammal tissue bank (MMMTB, www.marinemammals.eu) of the University of Padova, while the bovine tracheas were obtained from two cows at a local slaughterhouse. The MMMTB promotes the study and conservation of Cetacea. It is a CITES recognized research centre and tissue bank [7], supported by the Italian Ministry of the Environment and the University of Padova.

Details on the bottlenose dolphins used in this study are reported in table 1. Specimen condition indicates the physical state of the animal carcass on the date when the samples were obtained from the animal. The tracheas analysed in this study belonged to animals classified as grade 1 or 2 in a four-grade scale (1–4) as ‘fresh dead,' ‘moderate decomposition', ‘advanced decomposition' and ‘mummified'.

**Table 1.** Animal data and measures.

species	animal ID	sex	body mass (kg)	body length (cm)	specimen condition	ratio Sv/Sm (%)
*T. truncatus*	145	M	19	118	1	25.1
	159	M	261	328	1	21.1
	196	M	219	300	1	19.9
	319	M	300	310	2	18.0
*T. truncatus* mean ± s.d.						19.9 ± 4.1
*B. taurus*	1	F	≈450	n.a.	1	2.1
	2	F	≈450	n.a.	1	1.1
*B. taurus* mean ± s.d.						1.6 ± 0.5

The tracheas were removed and fixed by immersion in buffered formalin. They were cut into 10 mm transverse sections (*n* = 11 for *T. truncatus* and *n* = 8 for *B. taurus*), photographed and subsequently treated according to common procedures for paraffin embedding. Histological 5 µm sections were cut with a microtome and stained with haematoxylin–eosin. Images of histological samples were captured and examined using the slide scanner D-Sight 2.0 (Menarini Diagnostics, Italy). Morphometric analysis was performed to estimate the surface area of vascular lacunae (Sv) relative to the total surface area of the mucosa (Sm). The vascular contours and the total mucosa thickness were traced by two independent observers. The potentially confounding effect of formaldehyde on the calibre of vessels [8] is evened out in all specimens by using consistent and reproducible sampling and fixation procedures.

The results of the comparison of bottlenose dolphin and bovine average Sv/Sm ratios (±s.d.) are presented in table 1. In this study, the specimens belonging to the two groups (dolphins versus bovines) were compared with an *F*-test and a two tails *T*-test. *p* ≤ 0.01 was considered significant.

## 3. Results

### 3.1. Morphology

**Figure 1.**
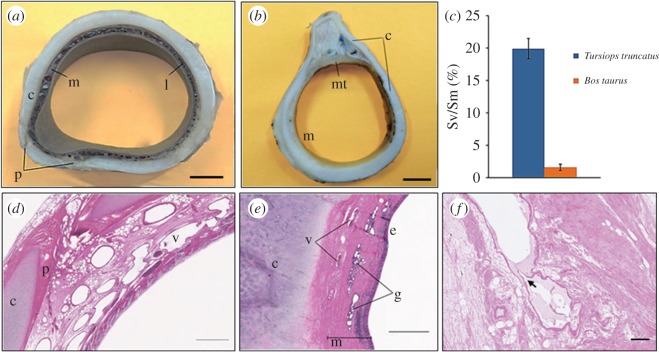
Photographs of tracheal sections of (*a*) *T. truncatus* and (*b*) *B. taurus*. (*c*) Differences (*p* < 0.01) in the Sv/Sm ratio between *T. truncatus* and *B. taurus*. Histology of tracheal sections from a bottlenose dolphin with large vascular lacunae (*d*) and bovine (*e*). In (*f*), the arrow shows a valve-like structure in a vascular lacuna of the bottlenose dolphin trachea. c, cartilage; m, mucosa; l, lacunae; p, perichondrium; mt, tracheal muscle; v, vascular lacunae; g, glands; e, epithelium. Scale bars: (*a*), (*b*) = 1 cm; (*d*) = 1 mm; (*e*) = 500 µm; (*f*) = 200 µm.

The examined tracheas of the dolphins and bovines correspond to the well-known anatomical description typical of the two species. Macroscopic cross-sections of dolphins' tracheas exhibit large vascular lacunae (figure 1*a*) that are absent in bovines (figure 1*b*). Observations of histological sections highlight the typical structure of the trachea. The lining of the trachea consists of a pseudostratified ciliated columnar epithelium that in some of the specimens is affected by post-mortem degradation processes. Hyaline cartilage is surrounded by a thick perichondrium, merging with loose connective tissue rich in adipocytes. A dense fibro-elastic connective tissue is present in the luminal part of the mucosa. The presence of large vascular lacunae is confirmed in the lamina propria-submucosa of the dolphin tracheas, while the corresponding part of the bovine tracheas only shows the presence of arteries and veins of medium to small calibre (see below), functional to supply the surrounding tissues (figure 1*d*,*e*). Occasionally, valve-like elements are observed in the large vascular structures of the dolphins' tracheal submucosa (figure 1*f*).

### 3.2. Areas

To evaluate the extent of vascular lacunae in tracheal sections, we measured the ratio between the sum of areas occupied by vascular structures (Sv) and the total surface of the mucosal layer, between the cartilage and the lumen (Sm). The percent ratios (Sv/Sm) are reported in table 1 and figure 1*c*. The difference between the mean Sv/Sm percentage in bottlenose dolphins (19.9 ± 4.1) and bovines (1.6 ± 0.5) was statistically significant (*F*-test: *p* = 1.2 × 10^−5^ < 0.01 different variance; and *T*-test: *p* = 2.2 × 10^−8^ < 0.01, different populations).

## 4. Discussion

The semi-rigid structure of the trachea of terrestrial mammals is functional to maintain constant airflow and, at the same time, to allow bending and partial rotation of the neck. Although whales and dolphins (except the beluga whale and river dolphins) possess less externally evident external neck mobility, the general architecture of the trachea remains the same, with the exception that the rings are continuous and intermingled. One of the specific differences, already noted in the early literature [9] and confirmed more recently [3,4], is the presence of peculiar features in the submucosal vasculature. However, no actual measurements or objective evaluations have ever been performed to understand whether the vascular supply and general blood vessel networks of the cetacean tracheal submucosa are just the result of individual anatomical and physiological plasticity [10], and, therefore, overextension of what may be found in terrestrial Cetartiodactyla, or a real adaptive trait derived from a specific evolutionary pressure. To this effect, we decided to compare the trachea of the bottlenose dolphin with that of the closely related *B. taurus*. In adult specimens of the two species, section areas of the tracheas were comparable, i.e. sections of the trachea of the same thickness have similar submucosal areas and volumes. Therefore, the need for vascular supply would be expected to be comparable. Furthermore, because cetaceans have no *m. trachealis*, and, therefore, lack the need for the relative blood supply, one would perhaps expect a less extensive vascular network. Microscopic analysis of the sections emphasized that the bovine does not possess the conspicuous vascular lacunae previously described in the bottlenose dolphin [4]. Our morphometric investigation indicates that the extension of the blood vessels, including the vascular lacunae, of the dolphin trachea is over 12 times larger than the corresponding structures in the bovine. Such a huge difference in surface points towards a specific innovative anatomical feature, and strongly suggests the hypothesis that the (said) lacunae may just represent a plastic adaptation.

We also emphasize that the presence of valves, detected in the dolphin lacunae, supports the existence of a passive mechanism of control of blood flow and a dynamic function for these structures. The direction and outcome of venous drainage of the lacunae in dolphins and cetaceans is currently unknown. In terrestrial mammals, venous flow from the trachea is mostly travelling towards the lower thyroid plexus (*v. thyroidea media* and *caudalis*) and subsequently into the *v. jugularis interna* [11]. However, considering the huge difference in the vasculature of dolphins, this route should be further investigated in detail and considered, together with the role of intramural innervation, in the complex of diving-related blood-shifts within the thorax (for discussion and reference, see [2]). Here, we emphasize that the veins of the dolphin trachea that either drain the lacunae or belong to the same vascular network, contain valves (figure 1*f*). The function of such structures is obviously related to prevent retrograde flow of blood. Considering that the conspicuous venous system of the trachea peculiar of dolphins and pygmy sperm whales is located within a rigid cartilaginous wall, the presence of valves further supports engorgement of the lacunae with consequent dynamic restriction of the lumen, for which effects a physiological explanation has been proposed in previous papers by our group [3,4]. Summarizing the different observations, a current hypothesis states that a reduction in volume of the tracheal lumen may result in increased pressure of the gaseous medium found in the lumen (air), and increases the robustness of the structure [4]. Further hypothesis has been proposed for the accessory air sinus of beaked whales [12], in which the thin-walled associated vascular network vessels, endowed with a large surface area, is likely to facilitate exchange of nitrogen gas and, therefore, form anatomic regions that may be important in the physiological management of diving gases.

The physiological advantage of an elaborate blood plexus in the trachea has not been fully explained yet. The complex dynamics of air flow during diving in dolphins and whales are beyond the scope of the present article. However, following the phenomenon of lung and alveolar compression, which takes place at approximately 235 m in human breath-holding divers [13] or at shallower depths in dolphins [10,14,15,16], air is trapped in the trachea and upper respiratory airways by constriction of the alveolar sphincters. Differently to what takes place in species that have a *musculus trachealis* (i.e. goats and by translation man), tracheal compression during descent is not complete in diving dolphins [4]. Previous reports suggested that the dolphin trachea only partially collapses under pressure [3,4] and then snaps back into shape with a certain velocity necessary to accommodate functional openings of the airways at the end of the post-dive ascent [4]. During this latter phase of the dive, partial engorgement of the vascular lacunae may improve resistance and adds velocity in the process of re-acquisition of the original form under conditions of decreasing environmental pressure [3,4]. Interestingly, venous valves have not been previously described in the systemic circulation of cetaceans, and we do not have enough elements to support their eventual role in the filling of the venous lacunae, perhaps through passive resistance to their voiding. Such a role would depend on the direction of the flow, impossible to determine based on the evidence available to us at the moment.

The precise mechanism by which the increase of the blood vessels constitutes an advantage for diving in these species is not fully understood or explained yet (for a recent review see [17]). Our present data indicate that such a mechanism may be the result of a specific evolutive tendency, possibly essential to increase the rigidity of the organ thus preventing baric damages during descent, and allowing air flow to move towards the larynx and nasal sacs for phonation and finally gas exchange at the surface.

## Supplementary Material

Epithelium of the dolphin trachea;Innervation of the dolphin trachea

## Supplementary Material

Epithelium of the dolphin trachea;Innervation of the dolphin trachea

## Supplementary Material

Epithelium of the dolphin trachea;Innervation of the dolphin trachea

## Data accessibility

Two images further supporting this article have been uploaded as part of the electronic supplementary material.
