# Supplementary material for: Vascularization of the trachea in the bottlenose dolphin: comparison with bovine and evidence for evolutionary adaptations to diving
Source: R Soc Open Sci. 2018 Apr 18;5(4):171645. doi: 10.1098/rsos.171645 (PMC5936905; doi:10.1098/rsos.171645)
Supplement: Epithelium of the dolphin trachea;Innervation of the dolphin trachea [file rsos171645supp1.docx]

Supplementary material – Legend of the supplementary Figures

Epithelium of the dolphin trachea

Cilia are evident in the pseudostratified epithelium of the trachea of the bottlenose dolphin. Blood vessels are present in the *lamina propria*, superficial to the deeper layer where the vascular lacunae are located. Scale bar = 100μm

Innervation of the submucosa in the dolphin trachea

Several (viscero-motor) nerve bundles (asterisk) travel in the connective tissue interspersed among the vascular lacunae of the trachea of the bottlenose dolphin. Scale bar = 100μm
