# Supplementary figures and images for: Vascularization of the trachea in the bottlenose dolphin: comparison with bovine and evidence for evolutionary adaptations to diving
Source: R Soc Open Sci. 2018 Apr 18;5(4):171645. doi: 10.1098/rsos.171645 (PMC5936905; doi:10.1098/rsos.171645)

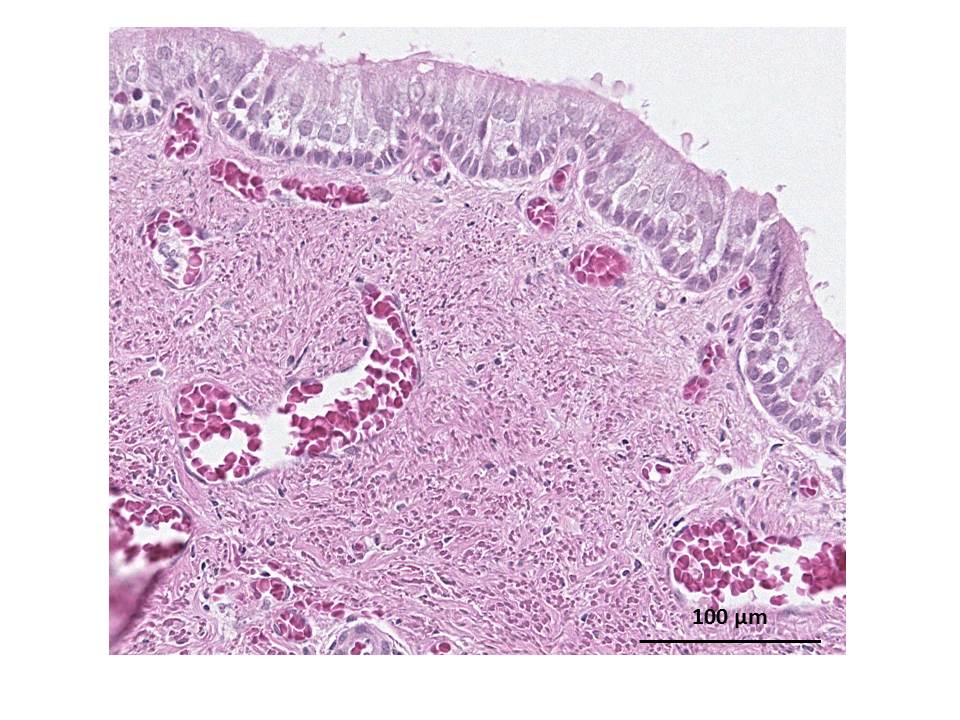

Supplement: Epithelium of the dolphin trachea;Innervation of the dolphin trachea [file rsos171645supp2.jpg]

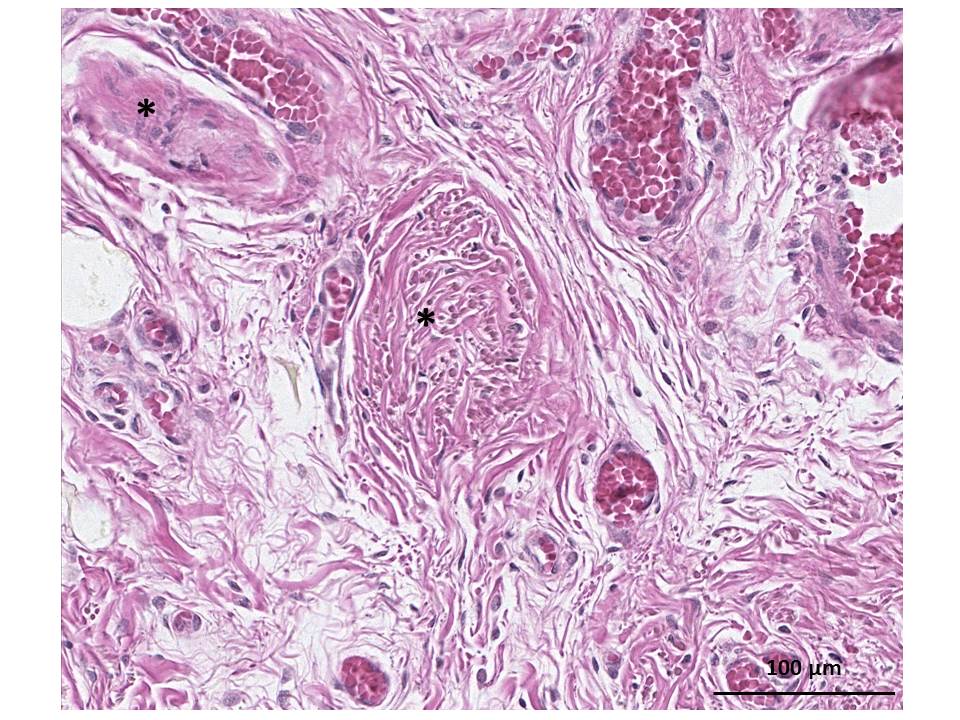

Supplement: Epithelium of the dolphin trachea;Innervation of the dolphin trachea [file rsos171645supp3.jpg]
